# Study of visfatin, asp level in obese children and their clinical significance

**DOI:** 10.1186/1687-9856-2013-S1-O40

**Published:** 2013-10-03

**Authors:** LI Rui-zhen, MA Xin-yu, LU Hui-ling, YAO Hui, CHEN Shou-kang, KANG Si-xiu, LIN Han-hua

**Affiliations:** 1 Department of Endocrine, Wuhan Children,s Hospital Affiliated to TongJi Medical College of Huazhong University of Science and Technology,Wuhan, Hubei Province,China; 2 Department of pediatrics,TongJi Hospital Affiliated to TongJi Medical College of Huazhong University of Science and Technology, Wuhan, Hubei Province,China

## 

To explore the relationship between visfatin, ASP and the incidence of childhood obesity and the significance of them in diagnoses and therapy of children obesity.

Eighty-six children (57 boys and 29 girls) including 40 obese children,22 overweight children and 24 normal control children were recruited with ages ranged 7-15 years. Serum visfatin and ASP levels were determined by ELISA.

(1) As compared with normal and overweight children, serum visfatin level was significantly higher in obese children (*p*<0.05);As compared with normal children (*p*<0.01)and overweight children (*p*<0.05), serum ASP level was significantly higher in obese children. There were no differences of serum visfatin and ASP levels between normal and overweight children. (2) The body mass index, TC, TG, LDL-c, FPG, FINS, insulin resistance index of the obese children were higher than the normal children (*p*<0.05 or *p*<0.01); the HDL-c and insulin sensitivity index of the obese children were lower than the normal group (*p*<0.01 or *p*<0.05). The body mass index, the fasting insulin, insulin resistance index of the obese children were higher than the overweight children (*p*<0.01). (3) Correlation Analysis: Serum visfatin and body mass index and TG were positively correlated (respectively r = 0.218, *p*<0.05; r = 0.500, *p*<0.01). ASP and body mass index, total cholesterol, triglycerides were positively correlated (respectively r = 0.268, *p*<0.05; r = 0.250, *p* <0.05; r = 0.427, *p*<0.01).

In simple obesity children, there were a significant change in serum visfatin and ASP, and visfatin and ASP involved in the disorder of lipids metabolism in obese children. Serum visfatin and ASP levels can be used as a new indicator to evaluate the risk of childhood obesity trends and future valuation of diabetes, cardiovascular.

